# A Multi-Level Iterative Bi-Clustering Method for Discovering miRNA Co-regulation Network of Abiotic Stress Tolerance in Soybeans

**DOI:** 10.3389/fpls.2022.860791

**Published:** 2022-04-07

**Authors:** Haowu Chang, Hao Zhang, Tianyue Zhang, Lingtao Su, Qing-Ming Qin, Guihua Li, Xueqing Li, Li Wang, Tianheng Zhao, Enshuang Zhao, Hengyi Zhao, Yuanning Liu, Gary Stacey, Dong Xu

**Affiliations:** ^1^Key Laboratory of Symbol Computation and Knowledge Engineering, College of Computer Science and Technology, Ministry of Education, Jilin University, Jilin, China; ^2^Department of Computer Science, Christopher S. Bond Life Sciences Center, University of Missouri, Columbia, MO, United States; ^3^College of Computer Science and Engineering, Shandong University of Science and Technology, Qingdao, China; ^4^College of Plant Sciences and Key Laboratory of Zoonosis Research, Ministry of Education, Jilin University, Jilin, China; ^5^Division of Plant Sciences and Technology, Christopher S. Bond Life Sciences Center, University of Missouri, Columbia, MO, United States

**Keywords:** miRNA co-regulation, abiotic stress tolerance in soybeans, homology expansion, bi-clustering, miRNA–target

## Abstract

Although growing evidence shows that microRNA (miRNA) regulates plant growth and development, miRNA regulatory networks in plants are not well understood. Current experimental studies cannot characterize miRNA regulatory networks on a large scale. This information gap provides an excellent opportunity to employ computational methods for global analysis and generate valuable models and hypotheses. To address this opportunity, we collected miRNA–target interactions (MTIs) and used MTIs from *Arabidopsis thaliana* and *Medicago truncatula* to predict homologous MTIs in soybeans, resulting in 80,235 soybean MTIs in total. A multi-level iterative bi-clustering method was developed to identify 483 soybean miRNA–target regulatory modules (MTRMs). Furthermore, we collected soybean miRNA expression data and corresponding gene expression data in response to abiotic stresses. By clustering these data, 37 MTRMs related to abiotic stresses were identified, including stress-specific MTRMs and shared MTRMs. These MTRMs have gene ontology (GO) enrichment in resistance response, iron transport, positive growth regulation, etc. Our study predicts soybean MTRMs and miRNA-GO networks under different stresses, and provides miRNA targeting hypotheses for experimental analyses. The method can be applied to other biological processes and other plants to elucidate miRNA co-regulation mechanisms.

## Introduction

The growth and development of crops are often restricted due to various environmental stresses, leading to poor harvests and yields below their genetic potential (Ku et al., 2015; Li et al., 2017). In the past decade, microRNAs (miRNAs) have been identified as important gene expression regulatory factors that play an essential role in plant growth and development (Ruiz-Ferrer and Voinnet, 2009). miRNA can target multiple genes, and multiple miRNAs can also target the same gene. miRNAs are involved in the expression of stress-responsive genes and the plant’s ability to adapt to environmental change (Sunkar et al., 2007). Different stresses can induce differential expressions of corresponding miRNAs in plants, while some miRNAs can simultaneously respond to several abiotic stresses (Shukla et al., 2008; Song et al., 2019; Sun et al., 2019). Therefore, studying the cooperative relationship among miRNAs and the interactions with their target genes is essential for understanding the role of miRNAs in controlling plant growth and development.

MicroRNAs may respond to adverse effects on plant growth and development, such as drought, salinity, temperature, and other abiotic environmental factors. It was shown that willow leaves exposed to drought or high temperature induce differential expressions of some miRNAs (Hivrale et al., 2016). For example, miR169c plays a negative regulatory role under drought stress by inhibiting the expression of its target gene nuclear factor Y-A (NF-YA) (Yu Y. et al., 2019). miR172a (Pan et al., 2016) and miR172c (Li et al., 2016) endow plants with a tolerance to salt stress and water deficiency. Meanwhile, miRNAs also indirectly respond to abiotic stress by regulating other biological macromolecules. For example, miR398c can negatively regulate multiple peroxisome-related genes (GmCSD1a/b, GmCSD2a/b/c, and GmCCS) and affect the drought tolerance of the soybean (Zhou et al., 2020). miR166k/o, miR390g, and miR396c/k mediate BX10 (Al-tolerant genotype) root elongation, and miR169r triggers the BD2 (Al-sensitive B genotype) oxidative stress, which in turn triggers a different type of plant aluminum tolerance between BX10 and BD2 (Huang et al., 2018). This indicates that miRNA may regulate plant growth under abiotic stress through a complex network. However, current studies typically explore the role of few miRNA in response to abiotic stresses. From a global view, how miRNAs work together as a co-regulatory mechanism has not been significantly explored.

Several studies have uncovered interesting miRNA interactions. For example, miR160 and miR167 are involved in the adventitious root program of Arabidopsis (Xu et al., 2014c). miR156 and miR172 play a role in the transition of soybean nutrition (Yoshikawa et al., 2013). Transgenic studies of miR482, miR1512, and miR1515 showed that their over-expression may lead to a substantial increase in the number of soybean nodules (Li et al., 2010). Another study verified networks of 365 tissue-specific miRNA–target interactions (MTIs) (Wang et al., 2019). In addition, Ismalia et al. (2019) used SVR to study the interaction between miRNA and lncRNA, constructed a network of miRNA–mRNA, miRNA–lncRNA, and miRNA–mRNA–lncRNA, and recognized their regulatory roles in stress response of *Arabidopsis thaliana*. Tu et al. (2022) mined the miRNA–lncRNA–TF regulatory network related to leaf and flower development of *Liriodendron chinense*, and pointed out that lch-lnc7374-miR156h-SPL3 and lch-lnc7374-miR156j-SPL9 are potential regulators of stamen and pistil development, respectively. And the miR157a-SPL and miR160a-ARF modules were validated using RLM-RACE, both of which are involved in leaf and flower development (Tu et al., 2022). The synergistic effects of miRNAs provide a new systematic perspective for the entire microRNome (Xu et al., 2014c), which calls for a global analysis of MTIs. Yang et al. (2021) found that the differential expression of key miRNA–target modules in plants may promote their root growth and development and enhance their tolerance to various stresses. Fu et al. (2019) revealed the response mechanism of potato miRNA–mRNA under alkali stress. It is of great significance to explore the biological mechanism of plants under abiotic stress from the perspective of miRNA–target.

Several methods have been developed and applied to explore this field with the growing miRNA-target data. Shalgi et al. (2007) first constructed a miRNA network from the target genes predicted by PicTar and TargetScan. Xu et al. (2011) constructed a human miRNA–miRNA functional synergy network through co-regulation functional modules. Meanwhile, biclustering was also applied for two different types of objects (gene and miRNA in this case) belonging to the same cluster. Various bi-clustering methods have been developed (Huang and Brutlag, 2001; Yoon and De Micheli, 2005; Caldas and Kaski, 2011; Xie et al., 2019). SAMBA (Tanay et al., 2002), ISA (Bergmann et al., 2003), BIMAX (Prelic et al., 2006), QUBIC (Li et al., 2009), and FABIA (Hochreiter et al., 2010) are some commonly used general algorithms. Contiguous column coherent (CCC) biclustering (Goncalves et al., 2009; Madeira et al., 2010; Medina et al., 2010; Goncalves and Madeira, 2014; Henriques and Madeira, 2014; Henriques et al., 2017) and LateBiccluster (Goncalves and Madeira, 2014) are designed for temporal data analysis. BicPAM (Henriques and Madeira, 2014; Henriques et al., 2017), BicNET (Henriques and Madeira, 2016) and MCbiclust (Bentham et al., 2017) are the latest tools. Pio et al. (2013) applied the biclustering algorithm to predict human miRNA–mRNA modules. The application of biclustering algorithms and miRNA–target regulation module (MTRM) mining is feasible and important for analyzing miRNA regulation mechanisms. Compared with traditional clustering methods, such as Bimax (Prelic et al., 2006) and BiBit (Rodriguez-Baena et al., 2011), CUBiBit (Gonzalez-Dominguez and Exposito, 2019) shortened the computing time and provided an optimized method for finding modules in larger data. However, the result obtained by CUBiBit was mostly a fully-connected bipartite graph, and the relationship between miRNA and the target gene is complex and interactive.

In this study, we proposed a method to obtain the miRNA regulatory modules and analyze their relationship in response to abiotic stresses in the soybean as a means for extending our understanding of soybean resistance mechanisms. Previously, Xu et al. (2014d) provided a soybean miRNA-gene network, SoyFN, based on predicted miRNA targets. However, this work was based only on sequence comparisons, which may result in a high false discovery rate. In contrast, in our work, we collected experimentally proven miRNA–target relationships based on degradome sequencing in the soybean and the stringent homologs of miRNA–target pairs in *A. thaliana* and *M. truncatula*. Based on these reliable miRNA–target data, we performed a biclustering analysis. We iteratively fused the overlapping biclusters based on the SoyNet network to obtain the soybean miRNA–target regulatory modules in response to abiotic stresses. We provide soybean MTRMs with high confidence relevant to various stresses, verified by REVIGO analysis to have the concentration of GO functions, and present the miRNA–GO regulatory networks of these modules. Capturing these miRNA–target modules with biological significance expands our understanding of the complex regulatory mechanisms of miRNA. The methods used should be readily applicable to other plant and animal systems where sufficient data exists to perform the analyses.

## Materials and Methods

**FIGURE 1:**
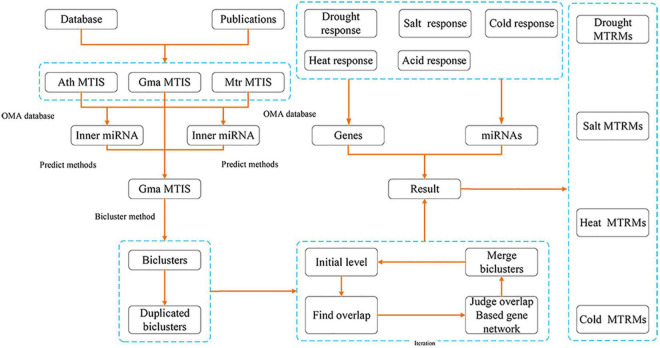
Flowchart of the authors’ research method.

We collected soybean MTIs from *A. thaliana* and *M. truncatula* databases and publications on miRNAs and genes of soybean response to several abiotic stresses. Subsequently, we used homology prediction on the collected MTIs to expand the soybean MTIs. Next, we used the biclustering method to mine the soybean MTRMs to perform overlap analysis to remove the redundancy. Then, based on the soybean gene interaction network, biclustering was applied through multi-level iteration. Finally, based on soybean abiotic stress-related miRNAs and genes, the fusion regulatory module was screened to obtain soybean abiotic stress-related MTRMs. Figure 1 shows a flowchart of our tasks and results.

### Data Collection

We collected miRNA–target data of *A. thaliana*, soybean and *M. truncatula* based on experimentally verified degradome sequencing results from databases [DPMIND, Tarbase, mirTarbase, and Starbase (Sethupathy et al., 2006; Hsu et al., 2011; Yang et al., 2011; Li et al., 2014; Vlachos et al., 2015; Fei et al., 2018)] and publications (Supplementary Table 1). In addition, we collected the miRNA information of the three species from the miRbase (Griffiths-Jones et al., 2008), the gene annotation of the species in the NCBI, EnsemblPlants, and the Phytozome (Goodstein et al., 2012; Howe et al., 2020). We also downloaded the homologous genes of *A. thaliana* and *M. truncatula* in Orthologous MAtrix (OMA) (Altenhoff et al., 2011). Besides, we downloaded the soybean cDNA sequence and soybean gene GO annotations from SoyBase (Grant et al., 2010), and obtained soybean gene network data from SoyNet (Kim et al., 2017).

We unified the miRNA and gene formats in the data in various databases and publications, then put the data of the same species together. Next, we annotated the miRNA–target data based on the collected and processed miRNA details and the gene annotations derived from the data of three species, including miRNA target data, related notes, and data sources. Finally, after processing the duplicated data, we obtained the miRNA–target data of the three species.

### Homologous Extension

We chose *A. thaliana* and *M. truncatula* to explore the potential targets. *A. thaliana* as a model plant has rich high-quality data. *M. truncatula* and soybean are closely related and have many similar biological characteristics. We extracted the miRNA sequence and removed redundant miRNAs with the same sequence in the soybean and *A. thaliana*. Subsequently, we extracted the target gene corresponding to the miRNA ID. Based on these targeted genes, we obtained soybean genes homologous to these genes from the *A. thaliana*-soybean homologous genes downloaded by OMA. We assumed that targeting relationships may exist if the sequences coexist and the genes are homologous. Therefore, these homologous genes may be targeted by these miRNAs in soybeans.

Targets obtained only based on homology information may not exist; so, we extracted these miRNA sequences and the cDNA sequence of target genes (SoyBase) and used miRNA-target prediction tools to predict potential relationships. We chose psRNAtarget (Dai and Zhao, 2011), TAPIR (Bonnet et al., 2010), and Targetfinder (Bo and Wang, 2005), whose results were better in non-Arabidopsis plants to predict potential soybean miRNA–target relationships (Srivastava et al., 2014). The three prediction software tools have different scoring methods. We analyzed their respective scores and merged them. The homology extension method for *M. truncatula*-soybean is the same as above.

### Clustering Method

The current research on miRNA targeting relationships is mainly based on one-to-one relative targeting. However, the miRNA targeting relationship is a complex interaction. The traditional clustering method is to cluster the same type of data, such as k-means, whose mining results in the miRNA-target regulatory module are poor because the targeting of miRNAs is sparse. The relationship between miRNA and the target gene is a bipartite graph structure; thus, the miRNA–target regulatory group can be found by analyzing the bipartite graph. CUBiBit (Gonzalez-Dominguez and Exposito, 2019) was proposed based on Bimax(Prelic et al., 2006) and BiBit (Rodriguez-Baena et al., 2011), which shortened the computing time and provided an optimized method for finding modules in larger data. We added the miRNA-target data based on the homology expansion predictions from *A. thaliana* and *M. truncatula* into the collected soybean miRNA-target data. Then, we extracted the miRNA-Target data with GO annotations and glyma2ID based on the soybean gene annotations of SoyBase. Finally, we used the CUBiBit to perform bi-clustering to obtain the results.

### Overlap and Iterative Fusion

**FIGURE 2:**
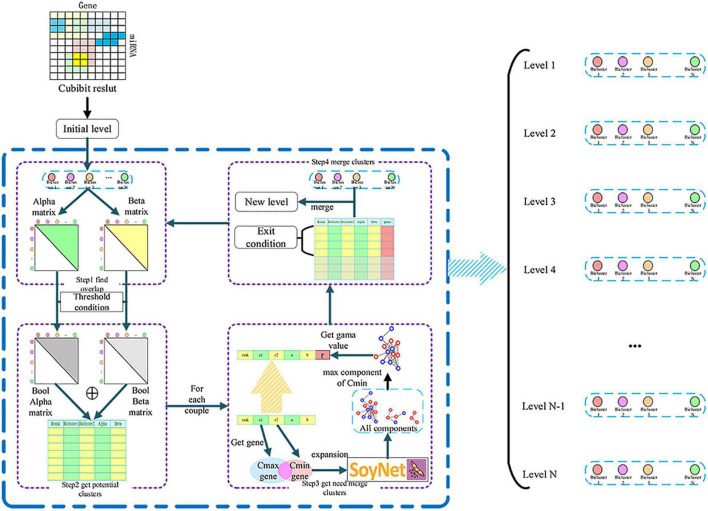
miRNA-target regulatory modules (MTRM) iterative merge algorithm flowchart used to derive a gene interaction network.

The result obtained by CUBiBit was mostly a fully-connected bipartite graph. However, the relationship between miRNA and target gene is complex and interactive. Therefore, we proposed a method of iterative fusion for MTRM modules based on a gene interaction network (Figure 2).

We detected the completely included classes in the clustering results and removed the included classes as the initial level result. First, for each class of this level containing miRNAs and genes, we judged the degree of overlap with other classes of miRNA and genes to form alpha and beta matrices, both of which are upper triangular matrices. After that, we set two thresholds of miRNA and genes that can be potentially merged for the two classes. We then recorded the two classes that met the potential fusion class-class table requirements to form a Boolean matrix. The initial alpha threshold was 0.3, and each iteration increased at a pace of 0.05 to conservatively determine the fusionable module and keep this value unchanged after rising to 0.8. It was sufficient if the beta threshold was greater than 0. Next, we extracted the union of two class genes and the network blocks of this pair of genes with a depth of 2 layers based on the SoyNet network for each pair of classes in the potentially merged class-class table. Subsequently, network blocks containing smaller classes were extracted from the obtained block set. We assumed that the network block with the most smaller class genes represented the function of the genes in the smaller class. Therefore, judging the number of genes in the major category of this network block can determine whether the genes of the two categories are similar in function. If the genes of the two classes were concentrated on a network block, which means that their genes interact closely and meet the conditions of potential fusion, the two classes can be merged. We compared the number of genes in the major category with the numbers of genes in all major categories in the sub-category function module to obtain scores and determine the correlation. Finally, we compared the number of genes in the major category with the number in all major categories in the sub-category function module to obtain scores to determine the correlation. The threshold was recorded as gamma. When gamma >0.3 was satisfied, the two classes were merged; otherwise, they would not be merged. For the class pairs that meet the fusion condition, we arranged them in descending order of alpha value and performed top-down non-repetitive fusion. Each class can only merge at most one class in one iteration. A new class set was formed as the new level, and the fusion result was the output. The next iteration would be performed and then another iteration until no fusion class pair could meet the two conditions.

### Function Assessment

Although this study does not include any experimental validation of our prediction, we assessed the distributions of gene functions indirectly to evaluate whether the results are biologically meaningful. For the results of the above iterative fusion, the enrichment of the classes in each level were separately analyzed. For a bicluster, we extracted its genes, used SoyBase’s GO BP and GO MF for enrichment analysis, and took the corrected GO ID with the smallest p-value as the best enrichment result for this type of cluster. When evaluating each class, the smallest p-value alone was not enough to assess the importance of the class. Instead, we used the cluster score to evaluate the enrichment of all the GO IDs enriched by the class. For all the enriched GO IDs of this class, we screened all the results with a *p*-value of less than 0.05 and then used Eq. (1) to calculate the cluster score of the class.


(1) $$\text{cluster}\,s\,c\,o\,r\,e=\frac{\sum_{1}^{n}\left(x_{i}-l\,o\,g\,\left(c\,o\,r\,r\,e\,c\,t\,P_{i}\right)\right)}{\sum_{1}^{n}x_{i}}$$

Among them, *n* is the number of gene ontologies enriched in the module, *x_i* is the number of genes enriched in the *i*-th GO, and correct*P*_*i*_ is the adjusted p-value of the *i*-th enriched GO.

### Abiotic Stress Response miRNA–Target Regulatory Module

We collected the miRNAs of soybeans that respond to drought, salt, acid, and low temperature based on our studies of publications (Subramanian et al., 2008; Kulcheski et al., 2011; Li et al., 2011; Sha et al., 2012; Subramanian, 2012; Sunkar et al., 2012; Dong et al., 2013; Zhang et al., 2014, 2018; Balyan et al., 2015; Xu et al., 2016; Zheng et al., 2016; Chen et al., 2018; Gupta et al., 2019; Proust et al., 2019; Yu J.-Y. et al., 2019; Wang et al., 2020). At the same time, we collected the differentially expressed soybean genes under various stresses. We screened these genes with foldchange ≥2 and *t*-test *p*-value less than 0.05 as related genes under abiotic stress. Then we marked the genes in the module and calculated the *p*-value related to abiotic stress based on the hypergeometric distribution. Finally, we screened based on the cluster score calculated by the module, the *p*-value related to stress, and the proportion of miRNA related to stress. In addition, the screening procedures related to drought and salt stress were consistent with the screening steps of the abiotic stress module.

### Construction of miRNA-Gene Ontology Network Under Abiotic Stress

**FIGURE 3:**
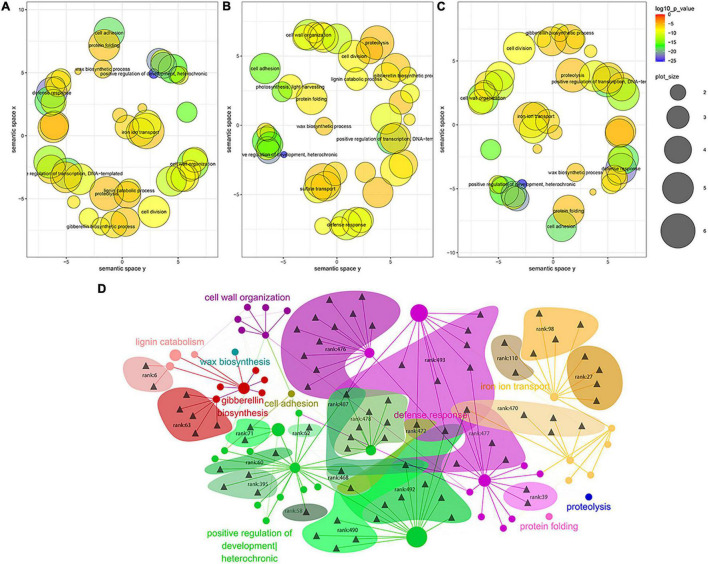
Gene ontology (GO) term analysis of MTRM genes under various abiotic stresses **(A–C)**. **(A)** GO semantic correlation analysis of abiotic stress, **(B)** drought stress, and **(C)** salt stress, and **(D)** the GO BP regulatory network of cooperative miRNAs under abiotic stresses. Triangles represent different miRNAs and circles represent different GOs. The size of the circle is determined by the number of genes contained in the GO in this article. The color of the circle depends on the representative GO. The areas with different colors show the modules obtained by our method.

Based on the results of MTRM mining under stress, we first screened the GO of the enrichment results in the screened module by *p*-value to remove the GO with a *p*-value less than 10^–5^; then, we performed a REVIGO semantic relevance analysis and extraction of concentrated representative GO channels. Based on the MTI data, the miRNA–GO relationship data was constructed through the gene pointed to by the miRNA in the module and the enriched go pathway to which the gene belongs. The relationship between GO is based on the results of REVIGO and the GO similarity calculation. The relationship is presented by setting a threshold to remove some weaker relationships. More detailed parameters are provided here or in the location of the specific figure (Figure 3D).

## Results

We obtained 90,064 confirmed soybean MTIs based on multiple experimental data sources and 1,189 potential soybean MTIs based on homology to experimental data from *A. thaliana* and *M. truncatula*. A multi-level iterative bi-clustering analysis resulted in 483 soybean miRNA-target regulatory modules and was evaluated according to GO enrichment function. In addition, we identified 37 abiotic stress-related modules and predicted the underlying miRNA regulatory pathway networks.

### Identification of miRNA-Target Interactions

We collected soybean miRNA–target data based on databases and related publications. First, we gathered all the soybean MTIs verified by degradome sequencing and biological experiments by mining published data. As a result, we obtained 111,650 pairs of soybean MTIs (Sethupathy et al., 2006; Song et al., 2011; Yang et al., 2011; Shamimuzzaman and Vodkin, 2012; Turner et al., 2012; Fang et al., 2013; Xu et al., 2013, 2014a; Ye et al., 2014; Yan et al., 2015, 2016; Chen et al., 2016, 2017; Ding et al., 2016; Liu et al., 2016; Fei et al., 2018), as shown in Supplementary Table 1. After removing 21,586 redundant pairs of MTIs, 90,064 pairs remained.

To expand MTIs, we predicted the target relationship between potential miRNAs and targeting genes from the MTIs of *A. thaliana* and *M. truncatula* based on homology. We obtained 12,094 unique pairs of Arabidopsis MTIs (Addo-Quaye et al., 2008; German et al., 2008; Ding et al., 2012; Xu et al., 2014b; Ma et al., 2018) and 4,394 unique pairs of Medicago MTIs (Devers et al., 2011; Lauressergues et al., 2012; Zhou et al., 2012; Ma et al., 2018) after removing redundant MTIs. Removing any redundant MTIs resulting from identical miRNA sequences, we further validated homology-based MTIs using three miRNA target prediction tools that performed well in general plants, i.e., psRNAtarget, TAPIR, and Targetfinder. In the Arabidopsis MTIs, a total of 961 unique pairs of MTIs were confirmed. In the Medicago MTIs, a total of 986 unique pairs of MTIs were confirmed, as shown in Supplementary Figure 1. There is a high overlap between the two sets of MTIs (Supplementary Table 2). After removing the redundant ones, a total of 1,189 pairs were used to expand soybean MTIs.

### miRNA-Target Regulatory Modules

We integrated the 90,064 soybean MTIs with the 1,189 MTIs based on homology. We removed MTIs involving genes that do not have the glyma2 ID. A total of 11,018 MTIs were removed, and the remaining 80,235 MTIs were used for analysis in the following tasks.

We applied CUBiBit for bi-clustering analysis, with the smallest scale 2 × 2 or 6 × 2 for miRNA-target modules (i.e., at least two or six target genes and at least two miRNAs in each module), resulting in 15,380 (2 × 2) miRNA-target modules or 2,461 (6 × 2) miRNA-target modules. We contracted the overlapping modules using a multi-level iterative fusion method based on the soybean gene relationship network (see section “Materials and Methods”), yielding 6,577 (2 × 2) and 812 (6 × 2) soybean miRNA-target regulatory modules after removing the modules that were completely included in the preliminary clustering module.

**FIGURE 4:**
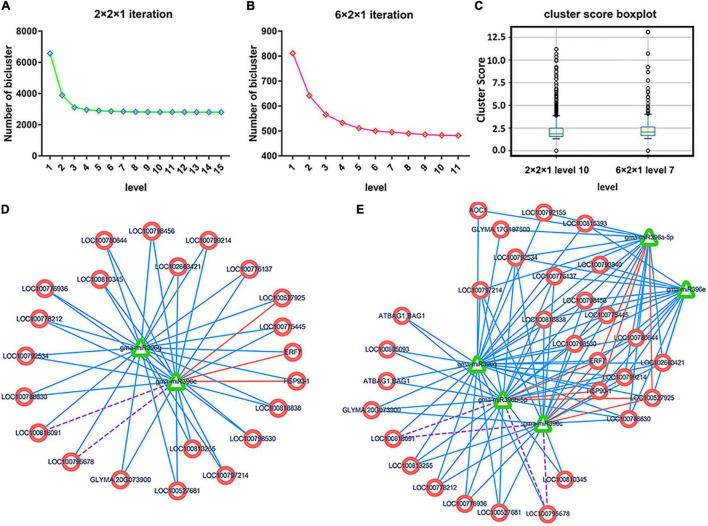
Multi-level iterative biclustering results of soybean MTRMs. **(A)** Results under different iteration times at the 2 × 2 scale, **(B)** results under different iteration times at the 6 × 2 scale, and **(C)** the boxplot of the cluster score is calculated based on the gene ontology (GO) under the two scales when converging to a stable level, where based on the overall distribution, the results at the 6 × 2 scale are better; **(D)** shows the MTRM bicluster at level 1 before the 6 × 2 scale fusion, and **(E)** shows the corresponding MTRM bicluster at level 7 after the 6 × 2 scale fusion.

We next merged MTRMs according to the set threshold until the level converged stably (level represents the number of iterations). Each level’s iterative fusion is shown in Figures 4A,B. We compared the iterative results at different scales. Soybean MTRMs at the 2 × 2 scale showed better results at level 10, which contains 2,715 MTRMs. Soybean MTRMs at the 6 × 2 scale showed a better effect at level 7, which contains 483 MTRMs. Comparing the cluster score based on the GO calculation between the two scales of stable convergence (Figure 4C) shows that the cluster score quality at the 6 × 2 scale is higher than that at the 2 × 2 level (Supplementary Table 3). Hence, we used the GO enrichment analysis result on 483 soybean MTRMs obtained at the 6 × 2 level 7.

To compare the MTRMs before and after fusion, we extracted an MTRM bicluster, as shown in Figure 4E, from the level 7 clustering results of the 6 × 2 scale and plotted it with the corresponding MTRMs under level 1 before the fusion, as shown in Figure 4E and after Figure 4D, which is a level-7 fusion. The module (1,534) is at level 1 before the fusion has 2 miRNAs and 22 targeted genes. At level 7, the module (1,534) fused an additional three modules, 1,539, 622, and 1,537, and each contains miR396. From the perspective of targeting, the module at level 7 has more miRNA-target interactions than the one at level 1.

### Gene Ontology Analysis and Evaluation of miRNA–Target Regulatory Modules

**FIGURE 5:**
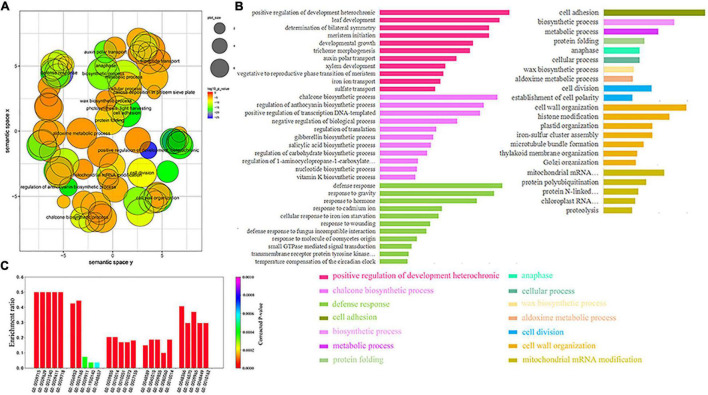
Gene ontology (GO) analysis of soybean MTRMs. **(A)** Semantic relevance of GO terms wherein the GO pathway has a certain concentration. **(B)** GO annotation enriched with 483 soybean MTRMs with enrichment results, which mainly involve positive regulation of development, heterochronic, chalcone biosynthesis, defense responses, and mitochondrial mRNA modification. **(C)** GO enrichment of the top five soybean MTRMs. The listed GO terms were enriched with significant *p*-values <0.00001.

We screened 254 GO pathways whose GO biological processes (BP) satisfied the *p*-value <0.00001 for the GO enrichment from 483 soybean MTRMs obtained at the 6 × 2 scale at level 7. We analyzed the relationship among the enriched GO terms through REVIGO (Supek et al., 2011) with a parameter of 0.5. These GO pathways have a specific aggregation (Figure 5A). MTRMs obtained from a global perspective have several concentrated distributions of GO functions, such as cellular processes, primary metabolism, cell adhesion, hormone response, and negative regulation of biological processes. In addition, there are metachronous positive growth regulations and chalcone biosynthesis. Chalcone plays an important role in soybeans and is involved in the multi-branch pathway of flavonoids and isoflavone biosynthesis (Subramanian et al., 2006). The enrichment results mainly involve positive regulation of development, heterochronic, chalcone biosynthesis, defense response, mitochondrial mRNA modification, sulfate transport, plant-type primary cell wall biogenesis, and cofactor biosynthesis, as shown in Figure 5B.

In addition, we extracted the enrichment results of the top biclusters in terms of cluster score among the 483 MTRMs and selected the top five GO terms of each module, as shown in Figure 5C and Supplementary Table 4.

### Abiotic Stress-Related Modules

**FIGURE 6:**
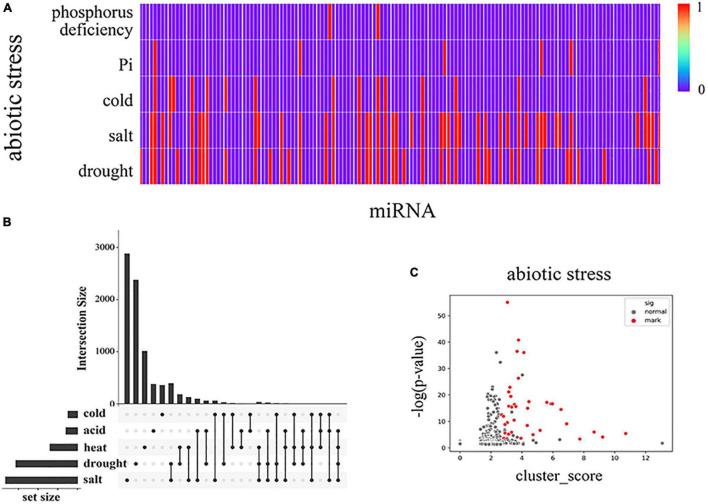
Collected miRNA data on soybeans involved in various abiotic stress responses based on the data statistics from the literature. **(A)** The distribution of stress types in miRNAs where each vertical line represents one miRNA, and red is marked as relevant, **(B)** UpSet diagram (Lex et al., 2014) of modular genes under various abiotic stresses within the horizontal correspondence, where dots are used to refer to the corresponding cold stress, acid stress, heat stress, drought stress, and salt stress on the left. The point-to-point connection is realized longitudinally to indicate the intersection between the corresponding data sets, and the upper bar graph shows the number of genes in the intersection. In panel **(C)**, the differentially expressed genes in each MTRM under abiotic stress are shown after screening. We used three indicators to filter the candidate clusters. According to the *p*-value, the related miRNA purity and the cluster score of each MTRM gene are placed under the corresponding stress. We selected the corresponding threshold, obtained the stress-related MTRMs with higher reliability, and marked them as red dots in Panel **(C)**. Supplementary Figure 2 shows MTRMs under other types of stress.

To explore the biological significance of soybean MTRMs, we collected related soybean miRNAs, which revealed five types of abiotic stresses, involving (1) drought, (2) salt, (3) cold, (4) Pi, (5) phosphorus deficiency based on publications (Subramanian et al., 2008; Kulcheski et al., 2011; Li et al., 2011; Sha et al., 2012; Subramanian, 2012; Sunkar et al., 2012; Dong et al., 2013; Zhang et al., 2014, 2018; Balyan et al., 2015; Xu et al., 2016; Zheng et al., 2016; Chen et al., 2018; Gupta et al., 2019; Proust et al., 2019; Yu J.-Y. et al., 2019; Wang et al., 2020). The function annotations of these miRNAs are shown in Supplementary Table 5. In most processes, soybean miRNA responses are involved in multiple abiotic responses, as shown in Figure 6A. Therefore, mining these miRNAs’ potential cooperative regulatory modules is important to understand their role in modulating soybean stress responses.

We correlated the 483 soybean MTRMs obtained by clustering with the functional annotations. We selected miRNAs that responded to drought resistance, salt resistance, heat stress, cold stress, and acid stress. And we performed a statistical analysis on the miRNAs in each of the 483 biclusters. We collected data on the differential expression of soybean genes in the MTRMs under drought, salt, low temperature, cold, and acid stress (Supplementary Table 6). The conditions for screening differentially expressed genes are log2FC > 1, *p* < 0.05. We obtained 2,145 differentially expressed genes under soybean drought and 1,752 differentially expressed genes under salt treatment. Figure 6B shows the genes in the module together with an abiotic stress diagram. At the same time, we calculated the *p*-values and FDR. We used the Benjamin Graham formula to correct the p-value of the genes in each MTRM for the differentially expressed genes under abiotic stress scenarios through the hypergeometric distribution, as shown in Figure 6C.

**FIGURE 7:**
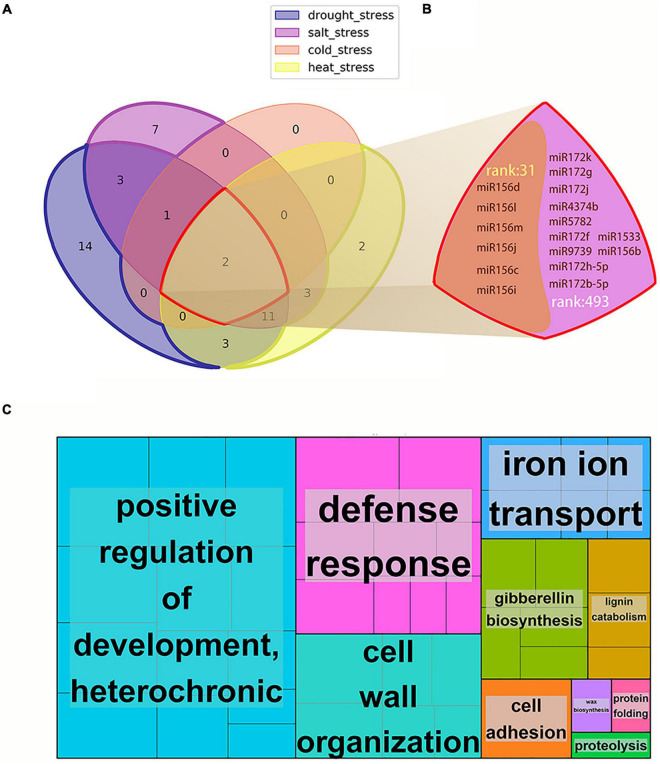
Soybean MTRMs under various abiotic stresses. **(A)** Venn diagram of 37 kinds of soybean MTRMs under various abiotic stresses, including 14 drought-specific MTRMs, seven salt-specific MTRMs, two heat-specific MTRMs, and two shared MTRMs, **(B)** the miRNAs in the two shared MTRMs 31 and 493, and **(C)** a GO Treemap of 37 MTRMs under abiotic stress.

Subsequently, we screened MTRMs related to abiotic stress, drought, and salt stress according to the *p*-value of differentially expressed genes corresponding to the stress in the MTRMs (*p* < 0.001, single adversity 0.01), the proportion of the corresponding miRNA family function (miR function ratio), and the cluster score (cluster score > median). The screening results are shown in Supplementary Table 6. We obtained 37 MTRMs related to abiotic stress, including 34 MTRMs related to drought stress, 27 MTRMs related to salt stress, 3 MTRMs related to cold stress, and 21 MTRMs related to heat stress. Figure 7A shows the set relationship of MTRMs involved in a variety of stresses. The data suggest that soybean miRNAs have basic and universal functional modules in their response mechanisms to drought, high salt, high temperature, low temperature, and other abiotic stresses. There are two shared modules (M31 and M493), involving 6 miRNAs and 11 miRNAs (Figure 7B), respectively. The six miRNAs of module M31 belong to the miR156 family. The regulated gene-enriched GO pathway is a transcription regulation, DNA-dependent (*p*-value 4.24 e-10), and a vegetative phase change regulation with a *p*-value of 9.24 e-07. The 11 miRNAs of module M493 are mainly in the miR172 family, in addition to miR156, miR1533, miR4374, miR5782, miR3939. The regulated gene-enriched GO pathway involves an oxidation-reduction process (*p*-value = 4.66 e-12) and a root hair elongation (*p*-value = 1.63e-08). Among them, miR156 is up-regulated in response to stress under drought conditions, in addition miR156d and miR156c play an important role in the heat tolerance of Arabidopsis (Zheng et al., 2016). miR172b, miR172h, miR172j-5p are down-regulated under drought stress to cope with water stress. miR156 is involved in the regulation of gene expression and signal transduction in response to soybean stimulation in a cold stress environment (Xu et al., 2016). miR156 and miR172 have been confirmed to respond to salt stress in a variety of plants (Sun et al., 2016). Moreover, we also found stress-specific regulatory modules in our results, including 14 drought-specific MTRMs, seven salt-specific MTRMs, and two heat-specific MTRMs (Supplementary Table 7).

The functions of related miRNA regulatory modules under abiotic stress are mainly concentrated in positive regulation of developmental heterochrony, defense responses, cell wall organization, and other biological processes, as shown in Figure 7C. In addition to plant positive regulation of development and defense response, GO functions such as cell wall organization also produce different response mechanisms under abiotic stresses. For example, salt stress disrupts cell walls integrity (Liu et al., 2021), and cell walls are adaptively regulate under drought stress (Moore et al., 2008). Moreover, plants reduce gibberellin production to reduce growth in order to concentrate energy against stress (Colebrook et al., 2014), sweet briar rose (*Rosa rubiginosa* L.) adapts to drought conditions by regulating gibberellin (Gadzinowska et al., 2020), and pea seeds adapt to heat stress by reducing gibberellin production (Leitão and Enguita, 2016), Gibberellin in *A. thaliana* is activated in a low-salt environment (Liu et al., 2013). Thus, GOs enriched in MTRMs play an important role in various stress responses. The data of the top five modules are shown in Supplementary Table 8.

### miRNA Regulatory Pathway Network Under Abiotic Stress

We explored the regulatory pathway network corresponding to the miRNA of the miRNA–target regulatory module in soybean abiotic stress and analyzed the GO terms of MTRM genes under various abiotic stresses. Stringent screening conditions were used, i.e., the *p*-value of MTRM stress is 0.001, and the GO BP pathway was selected with a *p*-value of less than 10^–5^. The REVIGO-based GO language correlation analysis is shown in Figure 3. GO channels with similar functions are closer in the distance in the figure. This is because the small RNAs targeting a certain cluster of GO are functionally close, and the 37 MTRMs of soybeans in abiotic stresses identified in this study mainly focus on resistance response, iron transport, positive growth regulation, and cell wall organization. Under abiotic stress, the cooperating miRNA regulatory modules of the soybean mainly regulate these pathways to respond to the stress environment. Figures 3B,C show the correlation analysis between drought stress and salt stress with specificity.

Subsequently, we constructed the GO BP regulatory network of cooperative miRNAs under soybean abiotic stress for the above main regulatory GO categories and miRNAs, as shown in Figure 3D. Multi-component miRNA families mainly regulate gene expressions related to abiotic stress responses. For example, the miR167 family regulates the resistance response pathway; the miR171 family regulates the gibberellin biosynthesis pathway, while the miR395 family participates in regulating the iron uptake. Moreover, some miRNAs have multiple GO functional partitions, such as miR156b, which regulates developmental growth, the timing of developmental events, the response to hormones, and the response to heavy metal cadmium. The miRNA families and regulatory pathways involved in MTRM are detailed in Supplementary Table 9.

## Discussion

miRNAs are major regulators of plant growth and development. They can also regulate environmental responses (Aukerman and Sakai, 2003; Chen, 2004; Zhu and Helliwell, 2011; Khraiwesh et al., 2012; Mao et al., 2013; Turner et al., 2013; Yan et al., 2013; Wong et al., 2014; Wang et al., 2015; Kulcheski et al., 2016). Hence, the study of the role of miRNAs is crucial—not only to understand the basic events of plant biology but to improve breeding for higher yields and more resilient crop plants. While various papers have noted the role of one or a few miRNAs in regulating plant stress responses, a global analysis of the cooperative interactions is lacking. To study miRNA regulation in response to abiotic response in the soybean, we collected a large number of soybean MTIs. In addition, we proposed a multi-level iterative fusion method of soybean MTRMs based on soybean gene networks.

We mined 483 soybean MTRMs, which provide a data reference for analyzing the cooperative miRNA mechanism of the soybean. Some MTRMs are involved in the biosynthesis of chalcone, which is derived from the general phenylpropanoid pathway that plays a wide variety of roles in soybeans and other plants. In most cases, gene regulation in each MTRMs involved a multi-component miRNA gene family. In some cases, these families were predicted to act cooperatively, which is consistent with the conclusion of Wang et al. (2019). And in the MTRMs we found under abiotic stress in soybean, such as regulatory module M477, which contains miR396, miR172, miR1507 and so on. Among them, soybean miRNA396 and miRNA172 are expressed in soybean drought (Zheng et al., 2016), and miR396s interact with growth-regulating factors (GRFs) to regulate plant growth, development and stress resistance. Liu et al. showed that 7 gma-miR396 (gma-miR396a/b/c/h/e/i/k) and 20 GmGRFs (GmGRF1/2/6-11/13-24) in soybean represent developed a many-to-many network interaction (Liu et al., 2017). Sahito et al. (2017) found that the expression level of soybean NNC1 (Nodule Number Control 1) affects its response to salt stress, while miR172 targets NNC1 and is induced by salt stress. In other plants, the expression of miR396 in rice and Arabidopsis affected the tolerance of plants under the saline-alkali stress (Ning et al., 2019), while another the expression of miR396 in rice was up-regulated under cold conditions (Sun et al., 2019). Sunflower HaWRKY6 (*Helianthus annuus*) gene expression is related to high-temperature stress, and miR396 has a regulatory effect on this gene (Giacomelli et al., 2012). It can be seen that miR396 has an important regulatory function under abiotic stress such as drought, cold, heat, and salt. Moreover, in the target proteins of the regulatory module M477, in addition to enzymes, transcription factors, etc., we also found some disease resistance-related proteins such as RPM1, RGA2, RGA, and some heat response-related proteins, DnaJ, heat shock 70 kDa protein 14, etc. Jiang et al. (2017) found that PWY-6842 was up-regulated in Arabidopsis under both biotic and abiotic stress. This also indicates that the regulatory mechanism of plants under abiotic stress may have commonalities between the underlying and biotic stress mechanisms. Recent studies have also shown that under biotic and abiotic stress, plants will have a series of signal regulatory networks, such as those mediated by Ca^2+^, ABA, and G proteins (Ku et al., 2018). The same miRNAs are differentially expressed in adversity (Kar and Raichaudhuri, 2021). It means that the MTRMs of soybean under abiotic stress we excavated have important significance for the regulatory mechanism of soybean under abiotic stress and the coordinated regulation of miRNAs.

Interestingly, we found that miRNAs from different families are also involved in the same regulatory gene clusters, which indicates that different miRNA families may have cross-family cooperative regulatory mechanisms in regulating certain functions. In contrast, miRNAs in the same family can be in different MTRMs; for example, the miR171 family (miR172b-5p, miR172h-5p, miR172f, miR172g, miR172j, and miR172k) are in multiple regulatory modules during drought and salt stress. Such hub miRNAs may be useful research targets for exploring soybean resistance mechanisms and resistance to breeding research under different stresses. After further combining the analysis of differentially expressed genes in soybeans under various stresses, we obtained the miRNA-GO regulatory network under abiotic stress. The GO BP contains a variety of important related pathways for understanding the common mechanisms in stress response. The research covering the plant miRNA regulation module can analyze the coordination mechanism of miRNA from a global perspective and determine the regulation relationship between modules, which may help explore the regulation mechanism of soybean miRNAs.

## Data Availability Statement

The original contributions presented in the study are included in the article/Supplementary Material, further inquiries can be directed to the corresponding authors.

## Author Contributions

HZ and DX conceived and designed the study. LS, Q-MQ, GL, XL, THZ, EZ, HYZ, and LW assembled the data. HC and TYZ performed the analyses and wrote the manuscript. HC wrote the modeling code. YL, HC, and GS assisted with interpreting the results. HZ, DX, and GS reviewed and revised the manuscript. All authors contributed to the article and approved the submitted version.

## Conflict of Interest

The authors declare that the research was conducted in the absence of any commercial or financial relationships that could be construed as a potential conflict of interest.

## Publisher’s Note

All claims expressed in this article are solely those of the authors and do not necessarily represent those of their affiliated organizations, or those of the publisher, the editors and the reviewers. Any product that may be evaluated in this article, or claim that may be made by its manufacturer, is not guaranteed or endorsed by the publisher.

## Supplementary Material

Supplementary Figure 1 Potential soybean MTIs predicted based on three prediction tools. **(A)** About 961 pairs of MTIs obtained based on the Arabidopsis data, **(B)** 986 pairs of MTIs obtained based on the Medicago data, and **(C)** 1,189 soybean MTIs from the union between panels **(A)** and **(B)**.

Supplementary Figure 2 The differentially expressed genes in each MTRM under other stress after the screening.

Supplementary Table 1 Summary of ATH, GMA, and MTR MTIs sources.

Supplementary Table 2 Potential targets found by homology expansion.

Supplementary Table 3 Comparison of the biclustering results of soybean MTRMs.

Supplementary Table 4 The top 5 GO enrichment of each top 5 soybean MTRMs.

Supplementary Table 5 miRNA function annotation.

Supplementary Table 6 Screening results of soybean MTRMs related to abiotic stress, drought and salt stress.

Supplementary Table 7 Stress-specific MTRMs and shared MTRMs.

Supplementary Table 8 Top 5 soybean abiotic response MTRMs.

Supplementary Table 9 GO terms of miRNAs in the soybean abiotic response MTRMs.
