# Supplementary figures and images for: A Multi-Level Iterative Bi-Clustering Method for Discovering miRNA Co-regulation Network of Abiotic Stress Tolerance in Soybeans
Source: Front Plant Sci. 2022 Apr 7;13:860791. doi: 10.3389/fpls.2022.860791 (PMC9021755; doi:10.3389/fpls.2022.860791)

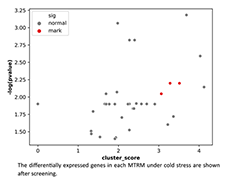

Supplement: Supplementary file 3 [file Image_3.TIF]

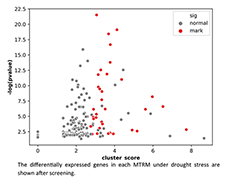

Supplement: Supplementary file 4 [file Image_4.TIF]

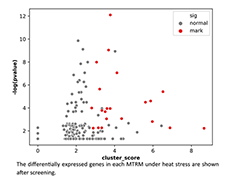

Supplement: Supplementary file 5 [file Image_5.TIF]

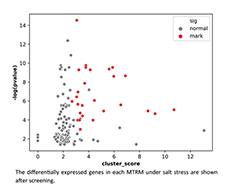

Supplement: Supplementary file 6 [file Image_6.TIF]

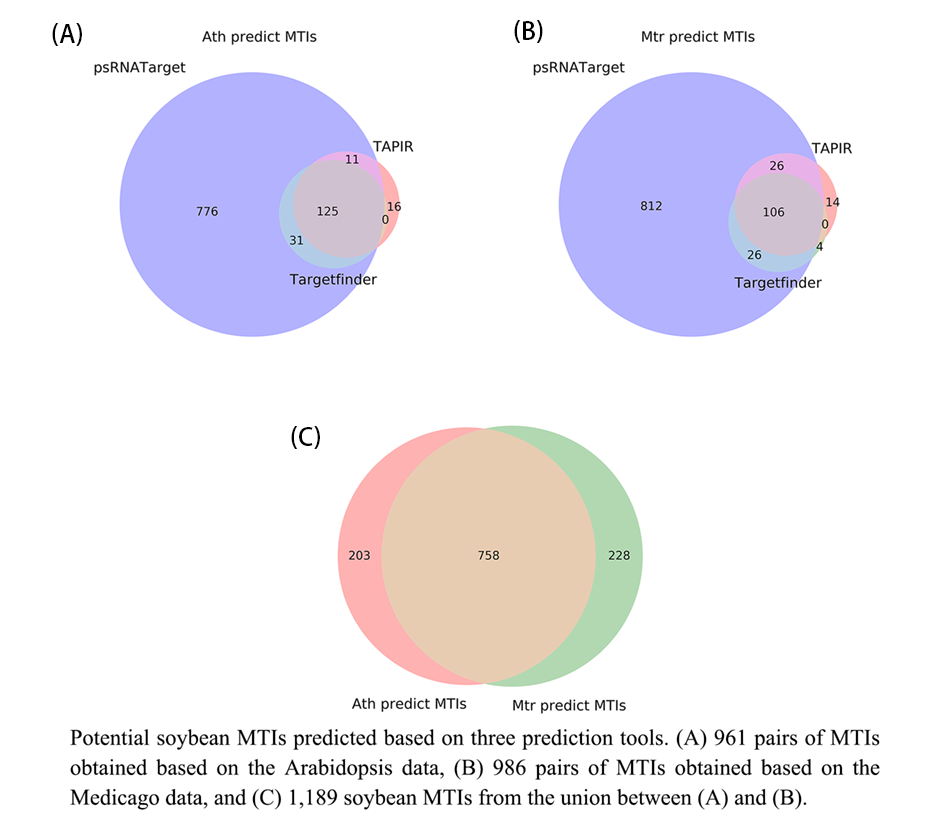

Supplement: Supplementary Figure 1 — Potential soybean MTIs predicted based on three prediction tools. (A) About 961 pairs of MTIs obtained based on the Arabidopsis data, (B) 986 pairs of MTIs obtained based on the Medicago data, and (C) 1,189 soybean MTIs from the union between panels (A) and (B). [file Image_1.TIF]

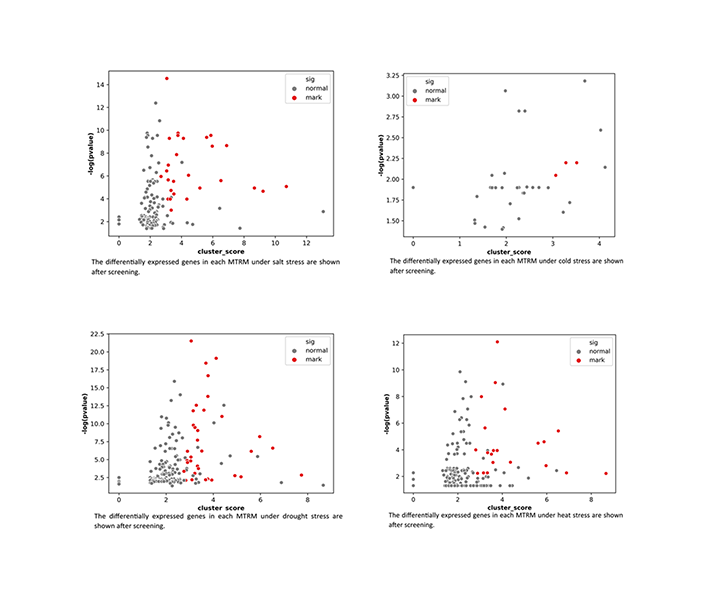

Supplement: Supplementary Figure 2 — The differentially expressed genes in each MTRM under other stress after the screening. [file Image_2.TIF]
